# Identification of NSTE-ACS patients with totally occluded infarct-related artery: the role of the SAVE risk score in improved risk stratification

**DOI:** 10.3389/fcvm.2025.1560369

**Published:** 2025-11-19

**Authors:** Biao Sun, Hong Liu, Lilan Ma, Tao Li, Jiaxin Shi, Xiaodan Yang, Guofei Ma, Xinhua Wu

**Affiliations:** 1 Department of Cardiology, The First Affiliated Hospital of Dali University, Yunnan, China; 2 Yunnan Trans-Plateau Cardiovascular Disease of Prevention and Treatment Research Center, Yunnan, China

**Keywords:** acute non-ST-segment elevation myocardial infarction, totally occluded infarct-related artery, electrocardiogram, risk stratification, SAVE risk score

## Abstract

**Objective:**

The aim of this study is to investigate the clinical characteristics of non-ST-segment elevation acute coronary syndrome (NSTE-ACS) patients with totally occluded infarct-related artery (IRA-TOCA) and validate the novel SAVE risk score for early identification.

**Methods:**

This retrospective study analyzed 185 consecutive NSTEMI patients undergoing coronary angiography, stratified into IRA-TOCA (*n* = 61) and IRA-non-TOCA (*n* = 124) groups. Baseline characteristics, angiographic findings, and post-percutaneous coronary intervention (PCI) biomarkers were compared. Risk stratification was performed using Global Registry of Acute Coronary Events (GRACE) and SAVE scores.

**Results:**

IRA-TOCA patients exhibited significantly higher post-PCI cTnI levels (8.3 vs. 3.34 ng/mL, *P* = 0.001), indicating more severe myocardial injury. Multivariable analysis identified IRA-TOCA [odds ratio (OR): 3.64, 95% confidence interval (CI): 1.77–7.49] and elevated brain natriuretic peptide (BNP) (OR: 1.001, 95% CI: 1.000–1.002) as independent predictors of cTnI elevation. The SAVE score demonstrated superior discriminatory ability (sensitivity 73.8%, specificity 54.8%; *P* < 0.001) compared to the GRACE score (*P* = 0.384). The left circumflex artery was the most common occlusion site (47.5%).

**Conclusion:**

IRA-TOCA represents a high-risk NSTE-ACS subtype with distinct biomarker profiles. The SAVE score enables early identification, potentially guiding timely revascularization.

## Introduction

Recent studies have indicated that approximately 70% of hospitalized acute myocardial infarction (MI) patients are diagnosed with non-ST-segment elevation acute coronary syndrome (NSTE-ACS), and this proportion is increasing annually (1). ST-segment elevation acute myocardial infarction (STEMI) results from complete coronary artery occlusion due to plaque rupture, erosion, and thrombosis, leading to sustained myocardial ischemia. In contrast, NSTE-ACS typically involves partial coronary artery occlusion, allowing for some degree of blood flow to the myocardium. Randomized controlled trials have demonstrated the efficacy of primary percutaneous coronary intervention (PCI) in reducing mortality and improving outcomes in STEMI patients (2). However, the role of early invasive strategies in NSTE-ACS is more nuanced and depends on the risk profile of the individual patient (3).

Surprisingly, even in NSTE-ACS patients, coronary angiography revealed that approximately 30% of the total occlusion of the infarct-related artery (IRA) was present, referred to as a totally occluded coronary artery (TOCA) (4). Current guidelines recommend that the management of NSTE-ACS focuses on antiplatelet therapy and anticoagulation to prevent further thrombus formation and progression. Invasive strategies, such as early PCI, are typically reserved for high-risk patients (5). Therefore, opportunities for early reperfusion therapy may be missed in those with IRA-TOCA due to the absence of classic STEMI ECG findings. However, this subgroup of patients demonstrates more severe clinical characteristics and worse prognosis compared to those with non-total IRA-TOCA (6, 7). Unfortunately, there is currently no reliable method to identify these patients early, before coronary artery angiography (CAG). The widely used Global Registry of Acute Coronary Events (GRACE) risk score (8), although valuable for general prognosis assessment, exhibits limited sensitivity in specifically identifying IRA-TOCA patients with a GRACE score of less than 140. This critical gap underscores the pressing need for a more targeted risk stratification tool, thereby highlighting the importance of developing novel risk assessment methodologies.

This study aims to address this gap by summarizing the clinical and coronary artery lesion characteristics of NSTE-ACS patients with IRA-TOCA, with a novel scoring system (9). We explore “STEMI-equivalent features” that can help clinicians identify patients with severe TOCA and guide timely revascularization to improve clinical outcomes.

## Materials and methods

### Study design and participants

**Figure 1:**
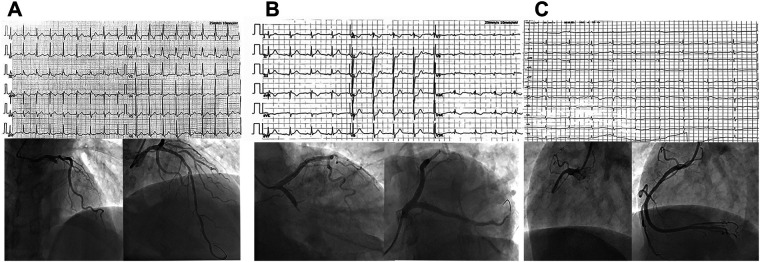
(**A**) A 62-year-old man was admitted to the hospital due to chest pain for 5 h. Electrocardiogram showed new onset right bundle branch block, manifested as rSR' pattern type in V1, QRS type in V5 and V6, broad S wave, QRS duration > 0.12S, but no ST elevation on anterior leads. Coronary angiography showed complete occlusion of the proximal segment of the LAD with 100% stenosis. (**B**) A 71-year-old man presented with chest pain for 4 h. Electrocardiogram showed ST-segment depression of 0.2–0.5 mV in leads V1–V4, especially in leads V2 and V3. Coronary angiography showed complete occlusion of the proximal LCX with 100% stenosis. (**C**) A 66-year-old man presented with recurrent chest pain for 1 week and aggravation for 12 h. Electrocardiogram showed ST-segment depression of 0.1–0.2 mV in leads II, III, and lead aVF. Coronary angiography showed complete occlusion of the proximal segment of RCA with 100% stenosis.

This retrospective study collected data from consecutive NSTE-ACS patients diagnosed by cardiologists at the First Affiliated Hospital of Dali University from September 2019 to January 2021. Patients underwent CAG and, if necessary, PCI. Based on CAG results, patients were divided into the IRA total occlusion group (IRA-TOCA) and the IRA-non-total occlusion group (IRA-NTOCA). Figure 1 shows typical ECG, angiographic, and PCI results. The Hospital Ethics Committee approved the study protocol.

Inclusion criteria for patients were typical chest pain symptoms lasting more than half an hour, 18-lead ECG without ST-segment elevation or pathological Q waves, elevated cTnI or elevated and then decreased cTnI, with at least one value exceeding the 99th percentile of the normal reference range, and CAG indicating at least one coronary artery with stenosis greater than or equal to 50%. Exclusion criteria were STEMI patients; myocardial infarction with non-obstructive coronary arteries (MINOCA) patients; patients with clear collateral circulation supply to infarct-related artery; patients with acute pericarditis, infective endocarditis, or viral myocarditis; patients with serious heart diseases such as rheumatic heart disease, alcoholic cardiomyopathy, valvular heart disease, or primary cardiomyopathy; patients with severe liver or kidney dysfunction, active bleeding, or autoimmune diseases; and patients with incomplete data.

### Baseline data collection

General information was collected, including gender, age, history of hypertension, diabetes mellitus, dyslipidemia, previous myocardial infarction, previous coronary artery bypass grafting (CABG), and smoking history.

### ECG measurement

All patients underwent ECG examination via an ECG machine to record 18-lead ECG (including 12 conventional leads and additional leads V7, V8, V9, V3R, V4R, V5R).

### Coronary angiography and result interpretation

The NSTE-ACS invasive treatment strategy risk assessment was used for risk stratification (10). Patients in the very high-risk group underwent urgent (<2 h) invasive treatment, those in the high-risk group underwent early (<24 h) invasive treatment, those in the moderate-risk group underwent delayed (<72 h) invasive treatment, and those in the low-risk group underwent non-invasive examination first. Cardiologists in our department performed coronary angiography through the radial or femoral artery approach, with PCI performed if necessary. According to the Schlesinger classification principle (11), coronary arteries were divided into three types: right coronary artery (RCA) dominance, balanced type, and left coronary artery dominance. The Rentrop classification (12) was used to evaluate coronary collateral circulation (CCC), with Rentrop grades 1, 2, and 3 indicating the presence of collateral circulation and Rentrop grade 0 indicating no collateral circulation.

### CTnI and BNP measurement

Following PCI, cTnI and brain natriuretic peptide (BNP) were measured using point-of-care testing (POCT), with the reference range for cTnI being 0–0.4 ng/mL and for BNP being 0–100 pg/mL.

### Echocardiography

All patients underwent color Doppler echocardiography during hospitalization, and left ventricular ejection fraction (LVEF) was recorded in detail.

### Ischemic risk assessment and risk stratification

The GRACE score is the most commonly used method for risk stratification of NSTE-ACS (13). A recent study by Tziakas et al. proposed a potential new method for identifying high-risk NSTE-ACS patients with TOCA, suggesting the use of the SAVE risk score (9). This score combines clinical, ECG, and echocardiographic findings to identify NSTE-ACS patients with IRA-TOCA. A SAVE risk score of ≥3 is considered high risk, while a score of <3 is considered moderate risk. The SAVE risk score system recommends that patients requiring urgent invasive treatment (<2 h) include those with STEMI or a new left bundle branch block (new LBBB), NSTE-ACS patients with very high-risk characteristics according to the GRACE score, and NSTE-ACS patients with a SAVE risk score of ≥3 but not meeting the very high-risk criteria of the GRACE score. Patients with a SAVE risk score of <3 are required to undergo invasive treatment within 24–72 h. In this study, all subjects were stratified using the GRACE score and the SAVE risk score.

### Statistical analysis

Statistical analysis was performed using IBM SPSS 26.0 (IBM Corp., Armonk, NY, USA). Continuous variables were assessed for normality using the Shapiro–Wilk test. Normally distributed data were expressed as mean ± standard deviation and compared using independent samples *t*-tests. Non-normally distributed data were expressed as median (interquartile range, IQR) and compared using Mann–Whitney *U*-tests. Categorical variables were presented as frequencies (percentages) and analyzed using Chi-square or Fisher's exact tests as appropriate. Correlation analyses were performed using Spearman's rank correlation coefficient (ρ) to assess relationships between post-PCI cTnI levels and clinical variables. Variables demonstrating significant associations (*P* < 0.10) in univariate analysis were included in multivariate stepwise logistic regression models to identify independent predictors of significantly elevated cTnI (defined as values > cohort median of 4.50 ng/mL). Results were reported as odds ratios (OR) with 95% confidence intervals (CI). Statistical significance was defined as two-tailed *P* < 0.05. All analyses adhered to STROBE guidelines for observational studies.

## Results

### Patient characteristics

According to the inclusion and exclusion criteria, the study cohort comprised 185 NSTE-ACS patients categorized into IRA-TOCA (*n* = 61) and IRA-NTOCA (*n* = 124) groups, with comparable baseline demographics including male predominance (82.0% vs. 79.0%, *P* = 0.639), similar mean age (61.6 ± 10.1 vs. 61.5 ± 10.3 years, *P* = 0.734), and equivalent cardiovascular risk profiles: hypertension (55.7% vs. 57.3%, *P* = 0.844), diabetes (19.7% vs. 25.8%, *P* = 0.357), dyslipidemia (29.5% vs. 35.5%, *P* = 0.418), smoking history (55.7% vs. 59.7%, *P* = 0.609), and prior myocardial infarction (1.6% vs. 4.0%, *P* = 0.673). Pre-admission medication use also showed no significant differences in antiplatelet agents (18.0% vs. 21.8%, *P* = 0.645), renin-angiotensin system inhibitors (19.7% vs. 24.2%, *P* = 0.372), statins (11.5% vs. 15.3%, *P* = 0.144), hypoglycemic agents (9.8% vs. 17.7%, *P* = 0.572), β-blockers (18.0% vs. 16.1%, *P* = 0.689), or calcium channel blockers (13.1% vs. 11.3%, *P* = 0.293). However, two critical differences emerged: symptom to first medical contact time was significantly shorter in the IRA-TOCA group (*P* = 0.024), and PCI was universally performed in IRA-TOCA patients versus 60.5% in IRA-NTOCA (*P* < 0.001), with no significant difference in first medical contact to angiography time (*P* = 0.207). These findings indicate that while traditional risk factors and baseline treatments were balanced between groups, IRA-TOCA patients presented earlier and received more intensive revascularization (Table 1).

**Table 1:** Comparison of the baseline characteristics between IRA-TOCA group and IRA-NTOCA group.

Risk factors	IRA-TOCA (*n* = 61)	IRA-NTOCA (*n* = 124)	*P*-value
Male (*N*, %)	50 (81.97)	98 (79.03)	0.639
Age (years, x ± s)	61.61 ± 10.09	61.53 ± 10.32	0.734
Hypertension, *N* (%)	34 (55.74)	71 (57.26)	0.844
Diabetes, *N* (%)	12 (19.67)	32 (25.81)	0.357
Dyslipidemia, *N* (%)	18 (29.51)	44 (35.48)	0.418
Smoking, *N* (%)	34 (55.74)	74 (59.68)	0.609
History of MI, *N* (%)	1 (1.64)	5 (4.03)	0.673
Medical history, *N* (%)			
Antiplatelet	11 (18.03)	27 (21.78)	0.645
ACEI/ARB/ARNI	12 (19.67)	30 (24.19)	0.372
Statins	7 (11.48)	19 (15.32)	0.144
Hypoglycemic agents	6 (9.84)	22 (17.74)	0.572
β-blocker	11 (18.03)	20 (16.13)	0.689
Calcium channel blocker	8 (13.11)	14 (11.29)	0.293
Therapy timeline			
Symptom to first medical contact [H,M (Q)]	5.0 (7.7)	9.4 (15.0)	0.024*
First medical contact to coronary angiography [H,M (Q)]	8.2 (7.9)	9.3 (10.7)	0.207
PCI, *N* (%)	64 (100)	75 (60.5)	0.000***

ACEI, angiotensin-converting enzyme inhibitors; ARB, angiotensin receptor blockers; ARNI, angiotensin receptor-neprilysin inhibitor (e.g., sacubitril/valsartan).

* *P* < 0.05; ***P < 0.001.

### Coronary angiography findings

The most common site of IRA-TOCA was the left circumflex artery (47.54%), followed by the left anterior descending artery (LAD) and RCA. The presence of collateral circulation, assessed using the Rentrop grading system, seemed to be higher in the IRA-TOCA (21.31%) group compared to the IRA-NTOCA (11.29%) group but with no significant difference (*P* > 0.05) (Table 2).

**Table 2:** IRA distribution between IRA-TOCA group and IRA-NTOCA group.

IRA Distribution		IRA-TOCA (*n* = 61)	IRA-NTOCA (*n* = 124)
LAD	Proximal	14 (22.95)	38 (30.65)
	Middle	4 (6.56)	14 (11.29)
	Distal	0 (0)	4 (3.23)
	Diagonal branch	2 (3.28)	2 (1.61)
LCX	Proximal	21 (34.42)	24 (19.35)
	Distal	6 (9.84)	10 (8.06)
	OM	2 (3.28)	2 (1.61)
RCA	Proximal	7 (11.47)	12 (9.68)
	Middle	2 (3.28)	3 (2.42)
	Distal	2 (3.28)	9 (7.26)
	PDA	1 (1.64)	0 (0)
LM	LM	0 (0)	6 (4.84)
Collateral circulation, *N* (%)		13 (21.31)	14 (11.29)

### Laboratory and imaging findings

Post-PCI cTnI levels were significantly higher in the IRA-TOCA group compared to the IRA-NTOCA group (*P* < 0.05). This finding is consistent with the more severe myocardial damage associated with IRA total occlusion. However, there were no significant differences in BNP levels and LVEF between the two groups (Table 3).

**Table 3:** LVEF, cTnI, BNP between IRA-TOCA group and IRA-NTOCA group.

Cardiac function	IRA-TOCA (*n* = 61)	IRA-NTOCA (*n* = 124)	*P*-value
LVEF (%), x ± s	58.70 ± 9.42	61.73 ± 10.91	0.066
cTnI (ng/mL), M (Q)	8.3 (20.60)	3.34 (7.55)	0.001**
BNP (pg/mL), M (Q)	131 (243.7)	124 (204.1)	0.528

** *P* < 0.01.

Univariate correlation analysis (Table 4) demonstrated that post-PCI cTnI levels exhibited significant positive associations with total occlusion status (*r* = 0.258, *P* < 0.001) and BNP levels (*r* = 0.254, *P* < 0.001), whereas no significant correlations were observed with hypertension (*r* = −0.094, *P* = 0.201), age (*r* = 0.078, *P* = 0.288), diabetes (*r* = −0.035, *P* = 0.643), gender (*r* = −0.107, *P* = 0.146), dyslipidemia (*r* = 0.004, *P* = 0.955), or smoking history (*r* = 0.065, *P* = 0.373). Subsequent multivariate logistic regression (Table 5) confirmed total occlusion (OR: 3.64, 95% CI: 1.77–7.49, *P* < 0.001) and elevated BNP (OR: 1.001 per 1-unit increase, 95% CI: 1.000–1.002, *P* = 0.017) as independent predictors of significantly elevated cTnI after adjusting for covariates. Notably, traditional cardiovascular risk factors, including hypertension (OR: 1.44, *P* = 0.271), age (OR: 1.01, *P* = 0.409), and diabetes (OR: 0.99, *P* = 0.979), showed no independent predictive value in the adjusted model.

**Table 4:** Correlation analysis of key clinical variables with cTnI levels.

Variate	*r*	*P*
Total occlusion or not	0.258	0.000***
BNP	0.254	0.000***
Hypertension	−0.094	0.201
Age	0.078	0.288
Diabetes	−0.035	0.643
Gender	−0.107	0.146
Dyslipidemia	0.004	0.955
Cigarette	0.065	0.373

*** *P* < 0.001.

**Table 5:** Binary multivariate logistic regression analysis of significantly elevated cTnI.

Variate	β-value	SE	Wald *c*^2^ value	*P-*value	OR	95% CI
Total occlusion or not	1.292	0.368	12.357	0.000***	3.642	1.772–7.487
BNP	0.001	0.000	5.651	0.017**	1.001	1.000–1.002
Hypertension	0.364	0.331	1.210	0.271	1.439	0.752–2.753
Age	0.013	0.015	0.682	0.409	1.013	0.983–1.044
Diabetes	−0.010	0.372	0.001	0.979	0.990	0.478–2.052
Gender	0.556	0.473	1.377	0.241	1.743	0.689–4.408
Dyslipidemia	−0.122	0.343	0.126	0.723	0.885	0.452–1.735
Cigarette	−0.260	0.361	0.516	0.472	0.771	0.380–1.566

** *P* < 0.01; ***P < 0.001.

### Risk stratification

**Figure 2:**
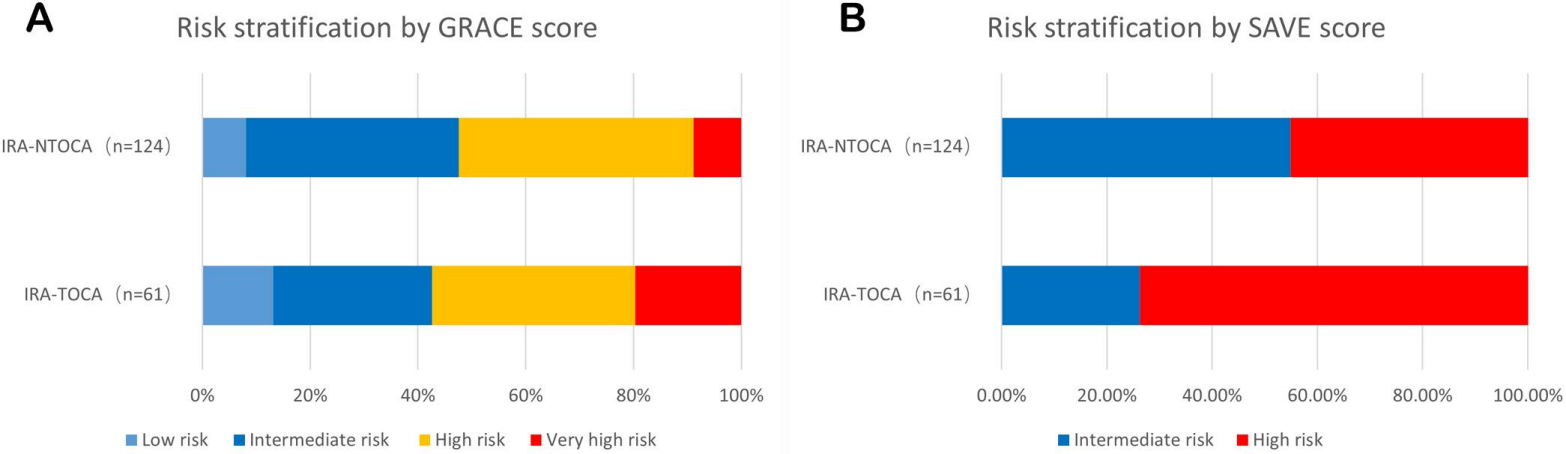
Comparative risk stratification by GRACE and SAVE scores. (**A**) Distribution of risk categories according to GRACE score in IRA-TOCA (*n* = 61) and IRA-NTOCA (*n* = 124) patients. Risk categories: low (<109), intermediate (109–140), high (>140), and very high (>140 with specific complications). No significant difference was observed between groups (*P* = 0.384). (**B**) Risk classification by SAVE score showed significantly higher proportion of high-risk patients (score ≥3) in IRA-TOCA group (73.8% vs. 45.2%; *P* < 0.001), with sensitivity of 73.8% and specificity of 54.8% for identifying IRA-TOCA. IRA-TOCA, infarct-related artery with total occlusion; IRA-NTOCA, infarct-related artery without total occlusion.

The GRACE score, a commonly utilized risk stratification tool for NSTE-ACS, did not differentiate between the IRA-TOCA and IRA-NTOCA groups (*P* > 0.05). This implies that the GRACE score may not be sufficiently sensitive for identifying NSTE-ACS patients with IRA-TOCA. In contrast, the newly proposed Survival (SAVE) risk score demonstrated significant differences between the two groups (*P* < 0.05). The SAVE risk score demonstrated a sensitivity of 73.77% and a specificity of 54.84% for identifying NSTE-ACS patients with IRA-TOCA, suggesting its potential value in clinical practice (Tables 6,7, Figure 2).

**Table 6:** Risk stratification by GRACE score.

Risk stratification	IRA-TOCA (*n* = 61)	IRA-NTOCA (*n* = 124)	*P*-value
Very high risk	12 (19.67)	11 (8.87)	
High risk	23 (37.71)	54 (43.55)	
Intermediate risk	18 (29.51)	49 (39.52)	
Low risk	8 (13.11)	10 (8.06)	
			0.384

**Table 7:** Risk stratification by SAVE score.

Risk stratification	IRA-TOCA (*n* = 61)	IRA-NTOCA (*n* = 124)	*-* value
High risk	45 (73.77)	56 (45.16)	
Intermediate risk	16 (26.23)	68 (54.84)	
			<0.001***

*** *P* < 0.001.

## Discussion

The increasing prevalence of NSTE-ACS underscores the need for improved risk stratification tools to identify the very high-risk patients who may benefit from immediate or early invasive strategies. Traditional risk factors for coronary artery disease may not be sufficient to predict IRA-TOCA in NSTE-ACS patients, emphasizing the importance of developing new risk assessment methods.

The present study demonstrated that approximately one-third of NSTE-ACS patients had IRA-TOCA, with the most common site being the left circumflex artery. This distribution aligns with previous studies (14–16), highlighting the higher prevalence of TOCA in the circumflex artery. These patients often delayed optimal invasive treatment, potentially leading to a poor prognosis.

A key strength of our study lies in the integration of clinical, biomarker, and angiographic data to develop and validate the innovative SAVE risk score, which showed higher sensitivity and specificity for early identification of NSTE-ACS patients with IRA-TOCA compared to the GRACE score. This integrative approach provides a readily applicable tool for bedside assessment, with direct potential clinical applications in early triage and cath lab prioritization for NSTE-ACS patients who are essentially experiencing an “occlusion MI” without classic STEMI criteria.

Even IRA-TOCA patients underwent earlier revascularization compared to the IRA-NTOCA patients; however, the time at which they received CAG was still much longer than that of patients with STEMI because of the indication of ECG. The lack of ST-segment elevation on ECG in NSTE-ACS patients with IRA-TOCA may be attributed to several factors. First of all, TOCA often involves the inferior and lateral walls of the left ventricle, and 12-lead ECG has lower sensitivity for detecting acute ischemia in these areas (17). Second, complete occlusion of the circumflex artery may lead to isolated posterior wall infarction, with ST elevation only observed in leads V7–V9 (18). Third, thrombosis in coronary arteries causing complete occlusion may resolve spontaneously or with medical treatment, resulting in transient ST-segment elevation that is not captured on admission ECG (19). Finally, if the infarct-related artery is congenitally small, complete occlusion of the distal segment may cause a minimal infarct area, leading to no ST-segment elevation on ECG (20).

Previous studies have shown that NSTE-ACS patients with IRA-TOCA have a higher incidence of cardiogenic shock and poorer clinical outcomes compared to patients with non-total occlusion (21). Our study also found significantly higher cTnI levels in the IRA-TOCA group, indicating more severe myocardial damage. The significantly elevated post-PCI cTnI levels observed in IRA-TOCA patients (8.3 vs. 3.34 ng/mL, *P* = 0.001) likely reflect procedural myocardial injury potentiated by total occlusion. We hypothesize two synergistic mechanisms: distal embolization of unstable thrombotic material during revascularization and ischemia-reperfusion injury in vulnerable myocardium with impaired microcirculation. This finding gains critical context from the recent study by Smith et al. (22), which demonstrated that periprocedural ischemic events independently predict 30-day mortality in NSTEMI (adjusted hazard ratio 2.3, 95% CI: 1.8–3.0). Our data extend this paradigm by suggesting that IRA-TOCA represents a morphological substrate for such injury. Future studies should evaluate protective strategies—such as deferred stenting or targeted thrombus aspiration—specifically in this high-risk subgroup.

The current guidelines for invasive treatment of NSTE-ACS are based on risk stratification. However, the identification and assessment of TOCA patients are challenging. Delayed invasive treatment for NSTE-ACS patients with IRA-TOCA may result in poor outcomes. Therefore, it is crucial to develop better risk stratification tools to identify high-risk TOCA patients and implement timely revascularization. The SAVE risk score proposed by Tziakas et al. showed promising results in our study, with high sensitivity and specificity for early identification of NSTE-ACS patients with IRA-TOCA. However, further clinical validation is needed before widespread application. The new classification of MI should be based on the presence or absence of acute coronary occlusion in the patient, rather than relying solely on ST-segment elevation on the ECG. This approach incorporates advanced ECG interpretation techniques, including artificial intelligence, bedside echocardiography, advanced imaging, and clinical signs of refractory ischemia, to more accurately identify occlusion MI patients (23).

This study has several limitations that warrant consideration. First, its single-center retrospective design and moderate sample size may introduce selection bias and restrict subgroup analyses. Second, the absence of short- and long-term clinical outcomes (e.g., mortality, heart failure readmissions, or major adverse cardiac events) prevents assessment of the prognostic utility of the SAVE score beyond acute biomarker profiles. Finally, the SAVE score requires validation in multiethnic cohorts with standardized endpoint assessment before clinical implementation can be recommended. Future studies should therefore adopt a prospective, multicenter design with a larger, more diverse population to improve generalizability, enable subgroup analyses (e.g., by age, sex, and comorbidities), and correlate SAVE scores with hard clinical endpoints and advanced imaging-based infarct quantification.

In conclusion, NSTE-ACS patients with IRA-TOCA are at high risk and require timely invasive treatment, characterized by a significantly elevated cTnI. The SAVE risk score may be a useful tool for early identification of these patients. Future research should focus on developing and validating new risk stratification tools to improve the management of NSTE-ACS patients with IRA-TOCA.

## Data Availability

The raw data supporting the conclusions of this article will be made available by the authors, without undue reservation.
